# SDA-Net: A Spatially Optimized Dual-Stream Network with Adaptive Global Attention for Building Extraction in Multi-Modal Remote Sensing Images

**DOI:** 10.3390/s25072112

**Published:** 2025-03-27

**Authors:** Xuran Pan, Kexing Xu, Shuhao Yang, Yukun Liu, Rui Zhang, Ping He

**Affiliations:** 1 College of Artificial Intelligence, Tianjin University of Science and Technology, Tianjin 300457, China; pxr@tust.edu.cn (X.P.); 22835026@mail.tust.edu.cn (K.X.); yangshuhao@mail.tust.edu.cn (S.Y.); liu3284671298@mail.tust.edu.cn (Y.L.); zruii@mail.tust.edu.cn (R.Z.); 2 School of Artificial Intelligence, Hebei University of Technology, Tianjin 300401, China

**Keywords:** remote sensing images, building extraction, multi-modal images, multi-scale feature fusions

## Abstract

Building extraction plays a pivotal role in enabling rapid and accurate construction of urban maps, thereby supporting urban planning, smart city development, and urban management. Buildings in remote sensing imagery exhibit diverse morphological attributes and spectral signatures, yet their reliable interpretation through single-modal data remains constrained by heterogeneous terrain conditions, occlusions, and spatially variable illumination effects inherent to complex geographical landscapes. The integration of multi-modal data for building extraction offers significant advantages by leveraging complementary features from diverse data sources. However, the heterogeneity of multi-modal data complicates effective feature extraction, while the multi-scale cross-modal feature fusion encounters a semantic gap issue. To address these challenges, a novel building extraction network based on multi-modal remote sensing data called SDA-les (AGAFMs) was designed in the decoding stage to fuse multi-modal features at various scales, which dynamically adjust the importance of features from a global perspective to better balance the semantic information. The superior performance of the proposed method is demonstrated through comprehensive evaluations on the ISPRS Potsdam dataset with 97.66% F1 score and 95.42% IoU, the ISPRS Vaihingen dataset with 96.56% F1 score and 93.35% IoU, and the DFC23 Track2 dataset with 91.35% F1 score and 84.08% IoU.

## 1. Introduction

Automated building extraction plays a vital role in supporting urban planning, environmental monitoring, and the development of smart cities by enabling efficient generation and updating of urban maps, significantly reducing the time and costs associated with manual map creation. Current building extraction methods primarily utilize convolutional neural networks (CNNs), Transformers, or hybrid architectures combining both, and mainly rely on optical remote sensing images [1,2,3,4]. However, single-modal optical remote sensing images have limited expressive capability and are prone to interference. By integrating multi-modal remote sensing data to complement each other’s advantages, overcoming the limitations of feature expression in single-modal optical data have become a key technological approach [5,6]. This cross-modal feature interaction significantly enhances target separability in complex scenarios, offering a new paradigm for improving building extraction accuracy. Several studies have explored different strategies for integrating multi-modal data. Teimouri et al. [7] used CNNs for building extraction from optical and SAR images, applying feature-level and decision-level fusion to improve the results. Li et al. [8] proposed a progressive fusion framework that merges common features from optical and SAR images through multi-level learning. Yuan et al. [9] introduced a CNN framework with an adaptive center-point detector, combining high-resolution aerial imagery and LiDAR data to address complex building segmentation challenges. Hosseinpour et al. [10] developed CMGFNet, utilizing a gated fusion module (GFM) to fuse RGB and DSM data, along with residual depth-wise separable convolutions to optimize the decoding process. Chen et al. [11] developed a cross-modal framework fusing remote and social sensing data to resolve low feature distinctiveness in urban village (UV) identification. Wang et al. [12] introduced the CUGUV dataset (thousands of UV samples across 15 Chinese cities) and a multi-source fusion framework, jointly addressing sample diversity and boosting cross-city UV mapping robustness. Yuan et al. [13] proposed a multi-scale semantic optimization network, achieving over 93.19% IoU accuracy by integrating cross-layer features from aerial imagery and LiDAR. In cross-modal segmentation, Li et al. [8] introduced MMFNet, a multi-stage fusion framework that improved building edge extraction accuracy by 9.5%. Tang et al. [14] developed ConTriNet, a triple-stream network using modality-specific flows to extract RGB/Thermal features and a complementary flow to fuse cross-modal cues, enhancing detection accuracy. Zhou et al. [15] proposed WaveNet, which employs wavelet MLPs for feature extraction and a Transformer teacher network to distill richer semantic–geometric knowledge into the student model. Wu et al. [16] designed CroFuseNet, which aggregates high-level features from optical and SAR data, achieving an MIoU of 0.9495 for impervious surface extraction. Li et al. [17] developed MSCDUNet, improving urban change detection accuracy by integrating multispectral, SAR, and VHR data using a multi-level heterogeneous fusion module, achieving optimal results on the MSBC dataset.

However, building extraction from multi-modal remote sensing data currently faces two major technical challenges [5,18]. First, the heterogeneity of multi-modal data leads to a significant gap in deep feature representations. The substantial differences in imaging mechanisms, spatial resolution, and noise distribution (e.g., speckle noise in SAR and spectral distortion in optical imagery) complicate the alignment of multi-modal feature spaces. Second, the inherent heterogeneity of multi-modal and multi-scale feature representations tends to exacerbate semantic discrepancies across different modalities and scales, which in turn significantly compromise the quality and effectiveness of feature fusion processes. Employing attention mechanisms to guide multi-scale feature fusion constitutes a widely adopted strategy for mitigating the adverse effects of multi-scale semantic gaps on feature fusion effectiveness [19,20,21,22]. However, prevalent attention mechanisms predominantly confined to single-channel or pixel-level weight computation exhibit critical limitations in global context integration, consequently degrading feature fusion performance through insufficient cross-regional dependency modeling. To address the above challenges, this paper proposes a novel building extraction framework, SDA-Net, with the following contributions:

(1) A Spatial Information Optimization Module (SIOM) is designed to effectively align spatial feature representations across different modalities. By leveraging feature modulation, decomposition, and reassembly, SIOM enhances multi-modal feature representation, bridges the gap between heterogeneous data, and reduces feature redundancy caused by early fusion.
(2) An Adaptive Global Attention Fusion Module (AGAFM) is proposed to intelligently guide multi-scale and multi-modal feature fusion. By modeling the dynamic relationships between spectral channels and spatial positions through global-adaptive attention mechanisms, AGAFM bridges semantic mismatches, balances local and global features, and generates high-quality fused features with improved discriminative capabilities for building extraction tasks.

Experimental results on ISPRS Potsdam dataset, ISPRS Vaihingen dataset, and DFC23 Track2 dataset show that the proposed method significantly improves accuracy in building extraction tasks under complex scenarios.

## 2. Related Work

Early automated building extraction techniques include Markov Random Fields (MRFs) [23] and object-based classification methods [24]. However, these approaches suffer from limited generalization capabilities, with performance significantly constrained in complex urban environments characterized by cluttered backgrounds, varying illumination, and diverse building morphologies. With the development of deep learning technology, deep convolutional neural networks have gradually become a key technology for remote sensing image interpretation [25,26,27,28] and this is also the case for building extraction tasks [29,30]. To further improve semantic segmentation capabilities, encoder–decoder architecture has been widely explored. Ji et al. [31] proposed a Siamese U-Net model with a shared-weight encoder–decoder architecture, improving building extraction accuracy, particularly for large buildings, and outperforming existing methods across multisource datasets. Feng et al. [32] designed a deep encoder–decoder network enhanced with superpixel conditional random fields (SCRFs), significantly optimizing building edge preservation. Hui et al. [33] proposed an end-to-end network architecture based on U-Net and designed an Xception module tailored for remote sensing images. The approach achieved promising results on the Massachusetts building dataset and the Vaihingen dataset. However, deep conventional architecture demonstrates structural limitations in effectively modeling global contextual relationships through sequential convolutional layer stacking for receptive field expansion. This approach not only manifests suboptimal efficiency in capturing long-range dependencies but also induces two critical drawbacks: the progressive diminishment of feature reuse capacity and systematic erosion of localized pattern preservation.

In recent years, self-attention and Transformer have been increasingly incorporated into building extraction networks to enhance models’ capability in capturing long-range contextual dependencies. Many works focus on self-attention mechanisms, leading to various innovative architectures. However, the high computational complexity of Transformer and conventional self-attention mechanisms has significantly constrained their applications in large-scale image processing tasks. To address this challenge, researchers have proposed various optimization approaches balancing performance and efficiency, primarily focusing on structured sparsification and computational pathway reformation. For the former, the Swin Transformer proposed by Liu et al. [34] introduces a shifted window mechanism that confines self-attention computation to non-overlapping local windows while enabling long-range dependency modeling through hierarchical cross-window feature fusion, substantially reducing computational overhead. Zhu et al. [35] introduced Bi-Level Routing Attention, which performs fine-grained local attention within fixed windows at the first level and dynamically selects critical regions via sparse routing for efficient global interactions at the second level. In computational pathway reformation, Ho et al. [36] developed Axial Attention, which decomposes 2D global attention into sequential single-axis computations along height and width dimensions, approximating global modeling through axial interactions. Han et al. [37] further proposed Agent Attention, employing lightweight agent tokens as information mediators to achieve linear complexity via a two-stage “aggregation-broadcast” operation while preserving global contextual awareness. These methodologies, leveraging structured sparsity and computational path reconfiguration, provide critical technical foundations for deploying Transformer architectures in high-resolution visual scenarios.

Meanwhile, numerous scholars have observed that although Transformers and conventional self-attention mechanisms exhibit superior perceptual capabilities, their serialized patch partitioning of input features disrupts the inherent spatial coherence of images. To address this limitation, hybrid architectures integrating Transformers and CNNs have been actively explored. For instance, Chen et al. [38] proposed TransUNet, which leverages self-attention mechanisms to encode tokenized image patches from CNN-generated feature maps into input sequences for global context extraction. He et al. [39] further developed ST-UNet, hierarchically integrating Swin Transformer’s global dependencies into CNN-derived features through a multi-scale architecture. Zhu et al. [40] introduced LMSwin_PNet, which optimizes the local information deficit of SwinTransformer through a local feature compensation module and multi-scale non-parametric attention. Diao et al. [41] designed MDTrans, a CNN-Transformer dual-branch parallel architecture to achieve cooperative extraction of local details and global context. Fu et al. [42] proposed CLGFF-Net, using a complementary feature fusion module (CFM) and a triple loss function to explicitly separate the shared and unique features of the convolutional and transformer branches. Sun et al. [43] developed FENET-UEVTS, which integrates a UNet encoder and visual transformer to enhance the robustness of irregular building change detection. These hybrid frameworks aim to synergistically combine the spatial preservation strengths of CNNs with the long-range modeling capacities of Transformers, addressing both structural integrity and contextual awareness in remote sensing image analysis tasks.

Accurate building extraction in remote sensing imagery demands multi-scale feature fusion to resolve scale diversity and structural intricacies. By integrating shallow-layer high-resolution details (edges, textures) with deep-layer semantic context (global layouts), this strategy concurrently addresses scale adaptation, occlusion mitigation, and cross-resolution consistency. It balances pixel-wise Precision with scene-level coherence, ensuring reliable segmentation across heterogeneous geographical scenarios. One approach leverages global attention for feature fusion, such as the Multi-head Attention Fusion Module (MAFM) proposed by Zhou et al. [44], which integrates multi-scale pixel-level and superpixel-level features but suffers from high computational costs. A more efficient alternative focuses on localized attention in specific dimensions, such as spatial or channel. For example, Fu et al. introduced DANet [45], which uses parallel spatial and channel attention to adaptively combine local features with global dependencies. Liu et al. [46] designed the Multi-Scale Attention Aggregation (MSAA) module, replacing skip connections in UNet to fuse multi-scale features through spatial and channel attention, enhancing feature representation. Further advancements refined these mechanisms. Jiang et al. [47] proposed a frequency-enhanced channel attention mechanism using Discrete Cosine Transform (DCT) to extract frequency information while minimizing high-frequency noise. Xiang et al. [48] integrated graph convolution theory into channel attention, developing an Adaptive Graph Convolution Module (AGCM) to adaptively learn feature group topologies, optimizing information extraction.

## 3. Methods

### 3.1. Overall Architecture

**Figure 1:**
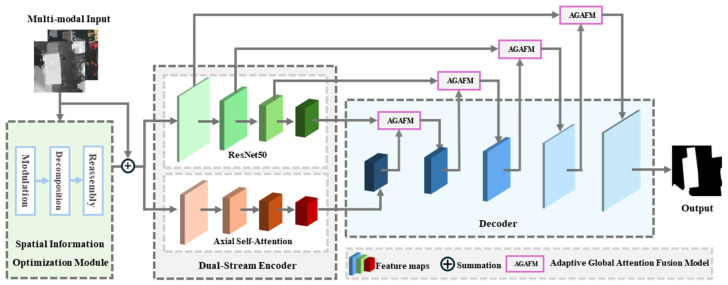
Overview of SDA-Net.

The SDA-Net, as illustrated in Figure 1, started with a Spatial Information Optimization Module (SIOM) which refines and aligns the multi-modal features through modulation, decomposition, and reassembly. The SIO initially modulates the multi-modal features from a multi-scale perspective, followed by the decomposition and reassembly of informative and non-informative features. This process enhances effective information and suppresses noise, thereby better aligning multi-modal features in the spatial dimension. The refined features are then input into the Dual-Stream encoder, which integrates ResNet50 [49] and axial self-attention [50] mechanisms in parallel. The axial self-attention mechanisms model global contextual information and extract complementary features from multi-modal data with less computational cost, while ResNet50 is focused on preserving the spatial details, compensating for any spatial loss that may arise from the self-attention mechanism. In the decoding phase, a progressive fusion strategy of encoder and decoder features is proposed, guided by the Adaptive Global Attention Fusion Module (AGAFM), to adaptively balance global and local multi-modal features. In AGAFM, the Adaptive Global Feature Adjuster (AGFA) is introduced to enable adaptive weighting and capture global dependencies effectively, thereby facilitating precise feature fusion at a fine-grained level. The fused features are gradually upsampled to the original image size, resulting in the final building extraction results.

### 3.2. Spatial Information Optimization Module

**Figure 2:**
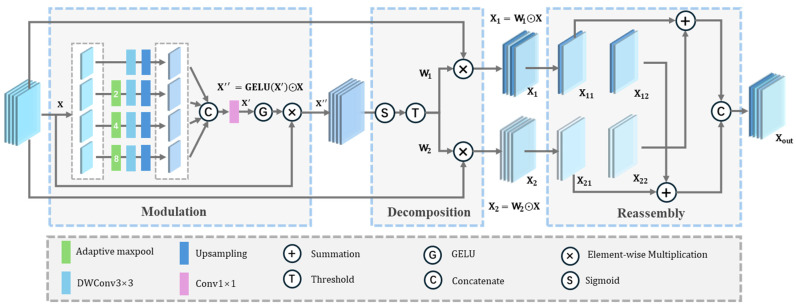
Spatial Information Optimization Module.

Different modalities of remote sensing data exhibit significant differences in spatial resolution, noise characteristics, and information content. Although early fusion strategies can integrate multi-modal inputs, they often lead to feature redundancy, thereby degrading the quality of extracted information. To address this, we propose a Spatial Information Optimization Module (SIOM) to enhance the multi-modal representations. As illustrated in Figure 2, SIOM operates through three sequential stages: Modulation, Decomposition, and Reassembly. In the modulation stage, multi-scale information is incorporated and selectively enhanced to adapt to the multi-scale geometric appearance of buildings. The decomposition stage employs gating mechanisms [51] to decompose informative features from redundant ones. Finally, in the reassembly stage, cross-channel interaction is performed to fuse the decomposed features, generating a consolidated representation with suppressed noise and enriched semantics.

The input feature is first enhanced through feature modulation. In this process, a multi-scale feature pyramid is introduced to incorporate multi-scale information into the feature representation, which helps to address the diverse geometric scales of buildings and the large intra-class variance. Specifically, the input $X\in {\mathbb{R}}^{H\times W\times C}$ is first divided into four groups along the channel dimension. To capture features at multiple scales, each group undergoes downsampling at different scales by adaptive max pooling operations, resulting in multi-scale features $X^{i}\in \;{\mathbb{R}}^{\frac{H}{2^{i-1}}\times \frac{W}{2^{i-1}}\times \frac{C}{4}}$, where $i=\left\{1,2,3,4\right\}$. To avoid premature fusion of multi-modal features, depth-wise convolutions are followed to extract spatial information at each scale. The multi-scale features are then upsampled to the original size and concatenated together. The 1 × 1 pointwise convolution is applied to fuse the combined feature and aggregate information across channels, enhancing feature expressiveness. The fused features $\;X^{\prime }\in {\mathbb{R}}^{H\times W\times C}$ are passed through a GELU activation function to generate an attention map, which reflects the importance of each position in the input features. By element-wise multiplication of the attention map with the original input features, the model is able to modulate the features according to multi-scale receptive fields, effectively enhancing the building features in the original multi-modal data, which can be expressed as follows:

(1) $$X^{\prime \prime }=\mathrm{GELU}\left(\;X^{\prime }\right)⊙X$$

In the decomposition stage, the importance of each spatial position is measured based on the grayscale distribution of the modulated features $X^{\prime \prime }$. $X^{\prime \prime }$ first passes through a Sigmoid activation function, mapping into the range [0, 1]. Based on a gating threshold *T*, the information-rich and informative-poor weight matrices $W_{1}$ and $W_{2}$ are generated. As shown in Formulas (2) and (3).

(2) $$W_{1}\left(i,j\right)=\left\{\begin{array}{c} \;1,\;\;\;\;\sigma \left(X^{\prime \prime }\left(i,j\right)\right)\geq T\\ \sigma \left(X^{\prime \prime }\left(i,j\right)\right),\;\;\sigma \left(X^{\prime \prime }\left(i,j\right)\right)<T \end{array}\right.$$

(3) $$W_{2}\left(i,j\right)=\left\{\begin{array}{c} \;0,\;\;\;\;\sigma \left(X^{\prime \prime }\left(i,j\right)\right)\geq T\\ \sigma \left(X^{\prime \prime }\left(i,j\right)\right),\;\;\sigma \left(X^{\prime \prime }\left(i,j\right)\right)<T \end{array}\right.$$

where (*i*, *j*) indicates the spatial position, and σ denotes Sigmoid function. Afterwards, $W_{1}$ and $W_{2}$ are element-wise multiplied with the input X, resulting in information-rich feature $X_{1}\in {\mathbb{R}}^{H\times W\times C}$ and the information-poor feature $X_{2}\in {\mathbb{R}}^{H\times W\times C}$:

(4) $$X_{1}=W_{1}⊙\;X$$

(5) $$X_{2}=W_{2}⊙\;X$$

where ⊙ denotes element-wise multiplication.

The information-rich feature $X_{1}$ and the information-poor feature $X_{2}$ are then cross-reassembled in the reassembly stage to generate an information-enriched feature $X_{\mathrm{out}}$. $X_{1}$ and $X_{2}$ are first split along the channel dimension into two parts, denoted as $X_{11}$, $X_{12}$ and $X_{21}$, $X_{22}$, and the shape of each subgroup is (*H*, *W*, *C*). The split features are then cross-reassembled and concatenated along the channel dimension as described in Formula (6).

(6) $$X_{\mathrm{out}}=\mathrm{Concat}\left(X_{11}+X_{22},X_{12}+X_{21}\right)$$

Overall, the proposed SIOM enhances feature representations through feature modulation, separates informative features via feature decomposition, and promotes channel interaction between information-rich and information-poor features through reassembly. This process enriches the data representation in the spatial dimension, facilitates the alignment of multi-modal data, and mitigates the feature redundancy caused by early fusion.

### 3.3. Adaptive Global Attention Fusion Module

**Figure 3:**
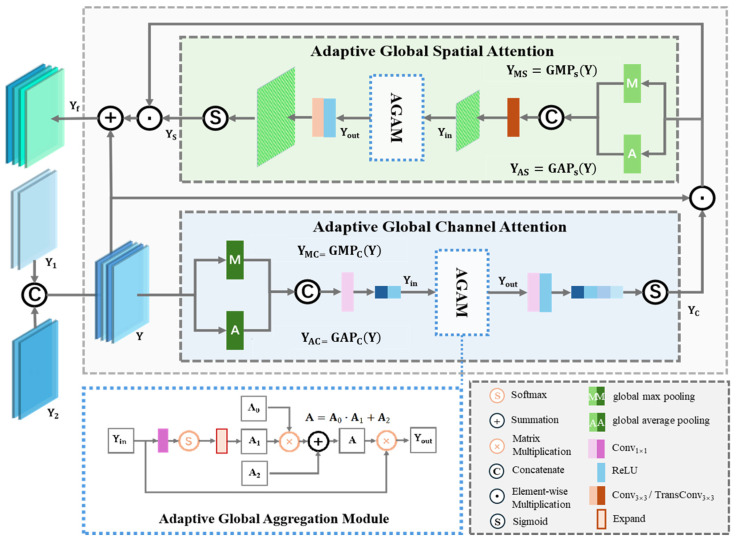
Adaptive Global Attention Fusion Module.

To more effectively guide encoder–decoder feature fusion, bridge the semantic gap in multi-scale multi-modal features, and optimally balance global–local information, we propose an Adaptive Global Attention Fusion Module (AGAFM). As shown in Figure 3, AGAFM is a cascaded spatial-channel attention mechanism that incorporates the Adaptive Global Aggregation Module (AGAM) during attention weight computation, endowing spatial and channel attention weights with genuine global contextual information. The sufficient aggregation of global information enables more effective semantic alignment and multi-modal alignment, thereby enhancing feature fusion efficacy.

The encoder feature **Y**_1_ and the decoder feature **Y**_2_ first undergo channel-wise concatenation → **Y** =Concat (**Y**_1_, **Y**_2_), and then feed forward to compute attention weights. Taking channel attention as an exemplar, **Y** undergoes spatial-wise global average pooling and global max pooling to generate **Y_AC_** and **Y_MC_**, respectively. These descriptors are concatenated along the channel dimension and fused via 1 × 1 convolutions, yielding the channel-wise salient- and overall-aware attention weights **Y_in_** as Equation (7):

(7) $$Y_{in}=\mathrm{Conv}\left(\mathrm{Concat}\left(Y_{AC}+Y_{MC}\right)\right)$$

However, **Y_in_** suffers from local receptive field limitations since its weights are computed through channel-wise operations that inherently neglect global channel dependencies. Inspired by reference [48], we proposed AGAM to refine the channel attention weights **Y_in_** through holistic contextual modeling across the entire feature map using graph convolution. AGAM constructs a graph structure by treating each element in **Y_in_**$\in {\mathbb{R}}^{C\times 1\times 1}$ as graph nodes, enabling comprehensive edge relationship learning between nodes through the definition of adjacency matrix **A** that encodes both geometric proximity and semantic similarity relationships. Specifically, **A** is an adjacency matrix with self-loops, formally expressed as follows:

(8) $$A=A_{0}\cdot A_{1}+A_{2}$$

where ⋅ denote matrix multiplication, and $A_{2}\in {\mathbb{R}}^{C\times C}$ denotes a learnable adjacency matrix, initialized with small values to ensure numerical stability during early training phases while maintaining gradient propagation efficacy. $A_{0}\in {\mathbb{R}}^{C\times C}$ is the identity matrix, and $A_{1}\in {\mathbb{R}}^{C\times C}$ is a diagonal matrix with normalized **Y_in_** values along its main diagonal, which serves as the self-loop weights in the graph structure. The adjacency matrix **A** then multiplied with **Y_in_**, implementing global-optimized refinement of the channel attention weights **Y_out_** as follows:

(9) $$Y_{\mathrm{out}}=Y_{\mathrm{in}}\cdot A$$

The final channel attention weights are generated through convolutional and ReLU layers and constrained within the interval [0, 1] through softmax normalization, as shown in Equation (10):

(10) $$Y_{C}=\sigma \{\mathrm{ReLU}[{\mathrm{Conv}}_{1\times 1}(Y_{\mathrm{out}})]\}$$

Similarly, the spatial attention weights $Y_{S}$ can be obtained by applying the same operations along the spatial dimension. The channel attention and spatial attention are sequentially applied to the features, followed by a residual connection with the original input **Y**. The final fusion result $Y_{f}$ is mathematically formulated as Equation (11).

(11) $$Y_{f}=Y+Y\;⊙\;Y_{C}\;⊙\;Y_{S}$$

where ⊙ denotes element-wise multiplication.

The AGAFM adaptively optimizes attention weights through global contextual aggregation during the encoder–decoder feature fusion phase, achieving superior multi-modal and multi-scale semantic alignment. This module provides optimized cross-modal features for multi-modal building extraction tasks.

## 4. Experiments

### 4.1. Datasets and Experimental Setup

**Figure 4:**
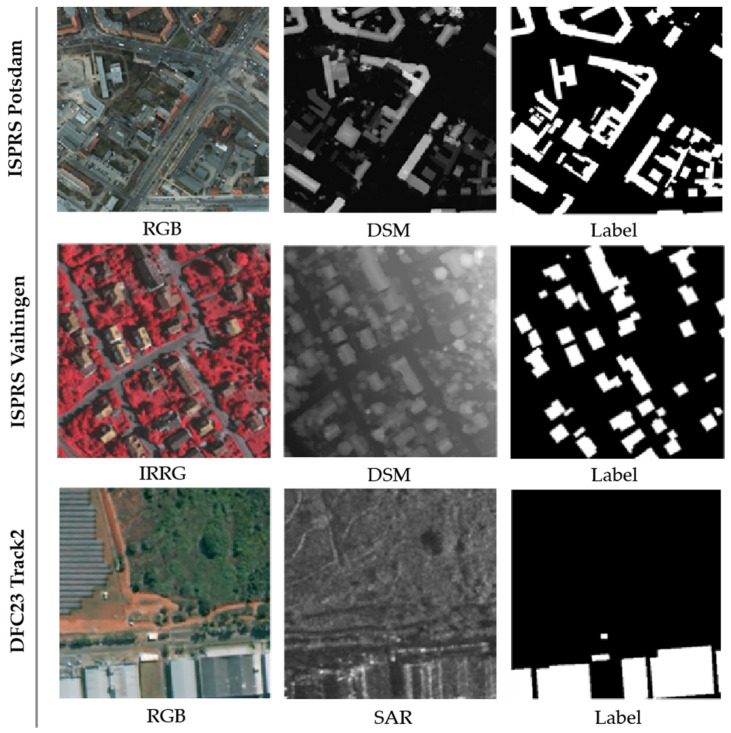
Example images from the ISPRS Potsdam dataset, ISPRS Vaihingen dataset and DFC23 Track2 dataset.

The experiments were conducted on the ISPRS Potsdam dataset, ISPRS Vaihingen dataset and DFC23 Track2 dataset. Figure 4 shows some sample examples from these datasets.

The ISPRS Potsdam dataset [52] contains 38 aerial images, each with a size of 6000 × 6000 pixels and a high spatial resolution of 5 cm. The images used in the experiments consist of the red, green, and blue bands (RGB) along with the corresponding digital surface models (DSMs). It is divided into six classes: impervious surfaces, buildings, low vegetation, trees, cars, and background. In the experiments, only the class building is considered.

The ISPRS Vaihingen dataset [53] consists of 33 true orthophoto (TOP) images with a spatial resolution of 9 cm. The experiment incorporates multi-spectral data comprising near-infrared, red, and green bands (IRRG) alongside corresponding digital surface models (DSMs). The dataset encompasses the same classes as Potsdam dataset, and our experiments also focus on class building.

The DFC23 Track2 dataset [54] consists of 1773 optical images of size 512 × 512 pixels and corresponding SAR images registered to the same geographic area. The optical images were acquired from two high-resolution satellites: SuperView-1 (0.5 m resolution) and Gaofen-2 (0.8 m resolution). The SAR data collection was performed using Gaofen-3 satellite with 1 m resolution capability. All SAR images were characterized by single-polarization configuration and underwent spatial resampling to maintain resolution consistency with the optical images.

The training was conducted on an NVIDIA GeForce RTX 3090 GPU, with input images cropped to 512 × 512 pixels and a batch size of 4. The initial learning rate was set to 0.0005, with cosine annealing employed for learning rate decay. The Adam optimizer was used to update the model weights during the optimization process. To reduce the risk of overfitting, data augmentation techniques, including random flipping, scaling, and color transformations, were applied to enhance the diversity of the training set and improve the model’s generalization ability.

The extraction results were evaluated using four metrics: Precision, Recall, F1 score, and Intersection over Union (IoU). Precision quantifies the accuracy of the model’s predictions, while Recall indicates its ability to capture all relevant targets. The F1 score provides a balanced assessment by combining both Precision and Recall, and IoU evaluates the spatial consistency between predictions and the ground truth. Together, these metrics offer a comprehensive evaluation of the model’s performance. By evaluating the proposed network across these datasets, its generalization capabilities in different environments and robustness in diverse scenarios can be thoroughly analyzed, ensuring more reliable building extraction capabilities.

### 4.2. Loss Function

In semantic segmentation tasks for remote sensing imagery, class imbalance is one of the core challenges affecting model performance. Specifically, building features typically occupy a smaller proportion of the entire image, while non-building backgrounds dominate the majority of the area. Traditional cross-entropy loss tends to optimize the dominant class, leading to insufficient recognition capability for minority classes. To address this issue, this study proposes a weighted loss function combining focal loss and Dice loss, which enhances the model’s sensitivity and segmentation accuracy for building targets through multi-objective optimization. The total loss function adopted in this study is the weighted sum of Focal loss and Dice loss, expressed as follows:

(12) $$\Delta J_{L}=0.5\cdot L_{\mathrm{fl}}+0.5\cdot L_{\mathrm{dice}}$$

where $L_{\mathrm{fl}}$ denotes Focal loss, and $L_{\mathrm{dice}}$ denotes Dice loss. The Focal loss formula is expressed as follows:

(13) $$L_{\mathrm{fl}}=-a_{t}{\left(1-p_{t}\right)}^{\gamma }\log\left(p_{t}\right)$$

where $a_{t}$ denotes the class weighting coefficient designed to balance the contributions of positive and negative samples, and γ serves as the modulation factor that reduces the loss weights for easy-to-classify samples. In our experiments, the parameters were selected as $a_{t}=$ 0.75 and γ=2.0, based on the class distribution of the dataset. Focal loss optimizes local hard samples, while Dice loss constrains global structures. Their synergy overcomes the limitations of single losses, effectively mitigates class imbalance, improves building boundary accuracy, and enhances model robustness in complex scenarios.

### 4.3. Comparative Experiment

#### 4.3.1. ISPRS Potsdam Dataset

To evaluate the effectiveness of SDA-Net in building extraction from multi-modal data, we conducted comparative experiments against several building extraction models on the ISPRS Potsdam dataset. These models include SERNet [29], RDFNet [55], REDNet [56], MMFNet [14], ADEUNet [23], SA-Gate [57], CMGFNet [10], TransUnet [39], and ST-Unet [40].

Quantitative comparisons listed in Table 1 reveal that the proposed SDA-Net achieves consistent improvements over existing methods on all performance indicators on the ISPRS Potsdam dataset. Particularly, our SDA-Net attains a Precision of 98.10% with 97.22% recall, outperforming existing methods in both metrics—concrete evidence of its advanced multi-modal representation learning. CMGFNet secures the second-highest performance metrics, exhibiting a marginal 0.18% deficit in F1 score and a 0.34% shortfall in IoU compared to the proposed SDA-Net. These comparative results underscore the operational efficacy of gating mechanisms in DSM-RGB fusion. Among other existing methods, SA-Gate and ADEUNet are competitive but still have limitations. SA-Gate’s feature decoupling aggregation mechanism suppresses noise but causes local feature dilution due to its bidirectional multi-step propagation, resulting in a 0.72% F1 score and 1.36% IoU gap compared to our method. ADEUNet’s joint spatial-channel attention mechanism improves feature fusion but its independent attention weights limit cross-modal synergy, leading to a 0.48% F1 score and 0.90% IoU reduction. Additionally, RDFNet exhibits the lowest Precision (5.00% lower) as a result of its early of multi-modal data, which fails to address the inherent discrepancies between modalities. RedNet demonstrates the poorest recall (6.99% lower) due to its inflexible hierarchical fusion approach, which is unable to effectively adapt to the multi-scale building features present in the Potsdam dataset.

**Figure 5:**
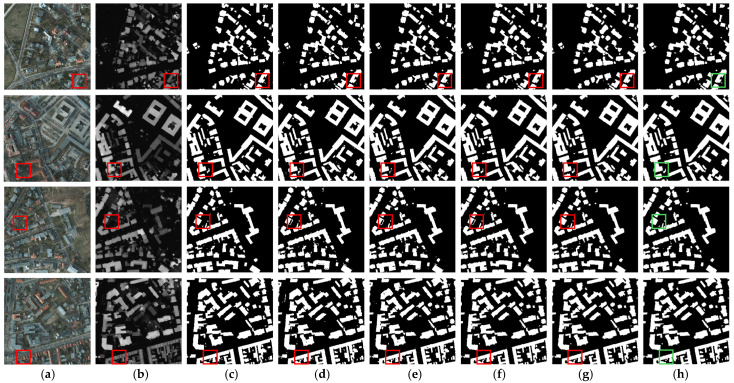
Visualization results on ISPRS Potsdam Dataset. (**a**) RGB images. (**b**) DSM images. (**c**) Labels. (**d**) SERNet. (**e**) RedNet. (**f**) ADEUNet. (**g**) CMGFNet. (**h**) SDA-Net.

The visualization results are depicted in Figure 5, demonstrating our method’s superior capability in mitigating feature redundancy within multi-modal data while achieving precise building identification in complex environmental conditions. As shown in the first two rows of Figure 5 (highlighted in boxes), buildings obscured by tree canopies create false-negative building extractions. All comparative methods are susceptible to interference from tree occlusion, exhibiting partial omissions in extraction results, while the accuracy of building contour delineation remains suboptimal. In contrast, our method effectively suppresses irrelevant interference through SIOM, yielding more precise building contour extraction. The third and fourth rows showcase representative scenarios where surface-level infrastructure (e.g., pavements, storage facilities) exhibits classification ambiguity with low-rise buildings. While comparative methods display varying degrees of misclassification, our proposed method significantly reduces such errors by leveraging advanced cross-modal complementary feature mining. This empirical evidence further substantiates the efficacy of AGAFM, which implements an adaptive global attention mechanism to achieve semantic alignment while maintaining equilibrium between global contextual awareness and localized feature preservation, thereby enabling comprehensive multi-modal feature exploration.

**Figure 6:**
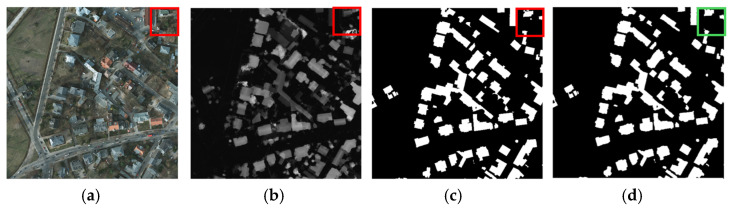
Failure case of the ISPRS Potsdam Dataset. (**a**) RGB images. (**b**) DSM images. (**c**) Labels. (**d**) SDA-Net.

As shown in Figure 6, a typical failure case is demonstrated. In the boxed area, the model fails to detect low-rise buildings with textures similar to their surroundings in optical imagery, primarily due to the loss of fine-grained features and insufficient capability to learn subtle spatial–spectral variations. During feature extraction, the model tends to overlook small-scale or low-contrast details, especially when targets share similar spectral and textural characteristics with their backgrounds. This indicates that our current approach still suffers from insufficient multi-scale feature fusion capability, where local details are often overwhelmed by dominant background patterns. An improved direction is to incorporate feature disentanglement techniques, such as wavelet transforms, to enhance local feature extraction by explicitly separating high-frequency details from low-frequency contextual information, thereby addressing the limitations of existing methods in preserving fine-grained structures.

#### 4.3.2. ISPRS Vaihingen Dataset

To comprehensively evaluate the performance of the proposed SDA-Net across varied scenarios, we extended the experiments to the ISPRS Vaihingen dataset, following the same experimental protocol as implemented on the ISPRS Potsdam dataset. As shown in Table 2, the proposed SDA-Net also achieves the best overall performance. Similarly to Potsdam dataset, CMGFNet, ADEUNet, and SA-Gate still achieved competitive results, with F1 scores differing by 0.60%, 0.49%, and 0.84%, respectively. This indicates that these methods exhibit certain robustness and can adapt to various types of multi-modal data and different urban scenarios. ST-Unet also demonstrates notable performance, achieving the second-highest recall score, which reflects the effectiveness of its Swin Transformer branch in integrating multi-modal data. However, its global–local feature fusion strategy via feature concatenation and channel-wise weighting remains limited, resulting in a 0.59% F1-score and 1.10% IoU gap compared to our method. In contrast, TransUnet, also a Transformer-based architecture, underperforms with a 1.89% F1-score and 3.48% IoU gap. This stems from its reliance on attention encoding over CNN-extracted features, where convolutional neural networks introduce excessive noise when processing multi-modal inputs, yielding lower-quality feature representations. SERNet, on the other hand, showed poorer performance, particularly with a significantly lower accuracy, falling behind by 6.45%. The observed performance limitation can be attributed to the repeated pooling operations within the global feature aggregation module, which likely induced progressive structural detail blurring and spatial information degradation in building boundaries.

**Figure 7:**
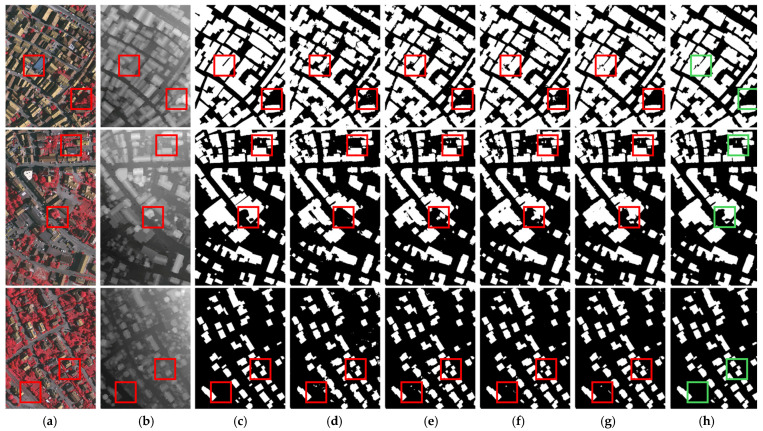
Visualization results on ISPRS Vaihingen dataset. (**a**) IRRG images. (**b**) DSM images. (**c**) Labels. (**d**) SERNet. (**e**) RedNet. (**f**) ADEUNet. (**g**) CMGFNet. (**h**) SDA-Net.

Some visualization results are shown in Figure 7, from which we can observe that shadow interference in the IRRG image and tree interference in DSM data may adversely impact building extraction results. Comparative methods demonstrate suboptimal performance in scenarios requiring suppression of interference signals within multi-modal data and effective retrieval of cross-modal complementary information. In contrast, our method optimizes multi-modal data representation through the SIOM and dynamically bridges the semantic gap via the AGAFM, achieving precise extraction results.

**Figure 8:**
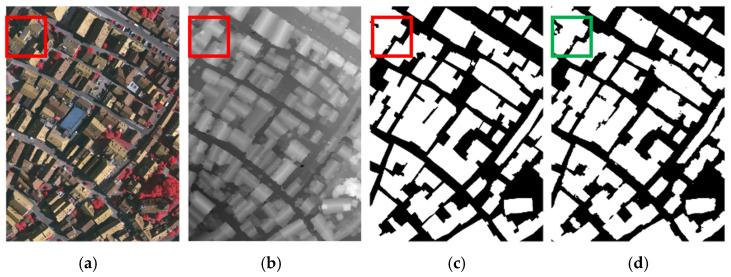
Failure case of the ISPRS Vaihingen dataset. (**a**) IRRG images. (**b**) DSM images. (**c**) Labels. (**d**) SDA-Net.

As shown in Figure 8, we analyzed a typical error case where the model incorrectly identifies shadowed regions as the background (boxed area). The primary causes are twofold: (1) texture feature degradation in RGB imagery due to shadow occlusion, which obscures critical roof surface details, and (2) ambiguity in DSM-based elevation features, where the shaded slope of inclined roofs exhibits bright patterns overlapping with low-elevation objects under elevation rendering algorithms. The root cause lies in the model’s inability to effectively reconcile texture loss in RGB imagery with elevation continuity in DSM, resulting in insufficient feature representation for shadowed regions. To address this issue, the improvement strategies include establishing a cross-modal compensation mechanism to enhance the model’s ability to recognize shadowed regions. Additionally, introducing geometric rule constraints further improves the recognition accuracy of shadowed areas. These methods effectively mitigate feature confusion caused by shadows and significantly enhance the robustness and accuracy of building extraction.

#### 4.3.3. DFC23 Track2 Dataset

To validate the building extraction capabilities of our SDA-Net across diverse data sources, we conducted experiments on the DFC23 Track2 (optical-SAR) dataset as well. The comparison experimental results are reported in Table 3, from which we can observe that SDA-Net exhibits superior performance across all evaluation metrics. Particularly, while maintaining the highest accuracy, SDA-Net significantly improves Recall compared to other approaches. Enhanced Recall indicates that our method, through feature modulation, decomposition, and reassembly of the SIOM, effectively addresses the interference caused by noise in SAR images during fusion, while emphasizing feature representation. MMFNet, which utilizes the phase as a modal invariant to jointly process optical and SAR images, effectively bridging the semantic gap and achieving the second-best performance. SA-Gate benefits from its feature decoupling aggregation mechanism, which partially alleviates noise interference in SAR data, resulting in good extraction performance. ST-Unet achieves commendable performance overall, yet its recall score exhibits a slight decline compared to the aforementioned experiments. This observation suggests potential limitations in its self-attention mechanism when handling SAR data characterized by higher heterogeneity. CMGFNet performs well in Precision but achieves the lowest Recall, indicating that its gating mechanism, designed to mitigate SAR noise interference, excessively suppresses feature expression.

**Figure 9:**
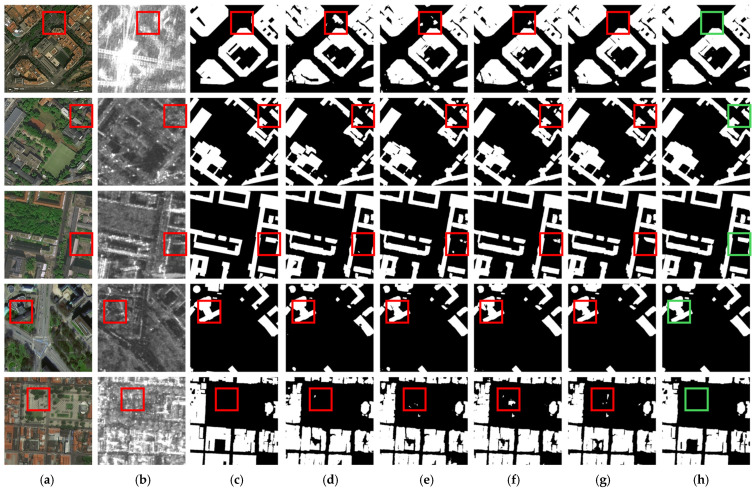
Visualization results of the DFC23 Track2 dataset. (**a**) RGB images. (**b**) SAR images. (**c**) Labels. (**d**) RDFNet. (**e**) RedNet. (**f**) SA-Gate. (**g**) MMFNet. (**h**) SDA-Net.

Figure 9 presents the visual analysis results, where the highlighted region in the first row demonstrates roads exhibiting textural similarity to building structures. Subsequent regions in the second and third rows capture buildings with environmental texture camouflage. Notably, the proposed method demonstrates robust discriminative capabilities in accurately extracting building contours under such challenging scenarios. The highlighted region in the fifth row illustrates a critical case where structural and material characteristics closely mimic building signatures amidst substantial SAR-specific speckle noise. The proposed SDA-Net effectively suppresses interference from irrelevant noise through adaptive feature modulation of the SIOM. While MMFNet achieves comparable performance in these areas, its cross-modal feature sharing mechanism constrained by phase invariance compromises the utilization of spatial and textural cues from optical data, particularly under suboptimal SAR imaging conditions.

**Figure 10:**
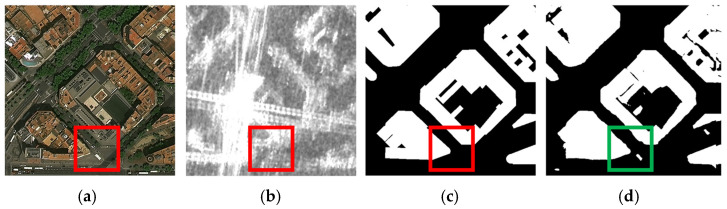
Failure case of the DFC23 Track2 dataset. (**a**) RGB images. (**b**) SAR images. (**c**) Labels. (**d**) SDA-Net.

The misclassification of vehicles as buildings observed in Figure 10 (boxed region) arises from the interplay of local feature ambiguities and deficient global contextual reasoning. Aerial view vehicles and compact architectural structures share overlapping visual traits in geometry, edge patterns, and spectral signature, while the model inadequately exploits spatial–semantic relationships with contextual elements such as road networks or adjacent vegetation. Furthermore, the limited spectral resolution of the image bands fails to capture critical reflectance distinctions between vehicle materials and building surfaces in diagnostically valuable wavelengths like near-infrared. To address these limitations, targeted enhancements include augmenting training datasets with densely packed vehicle scenarios (e.g., parking lots) to improve feature robustness, integrating road vector data for spatial constraint modeling, leveraging multi-spectral analysis to amplify material-specific reflectance disparities, and recalibrating loss functions to prioritize boundary-sensitive optimization.

### 4.4. Ablation Study

#### 4.4.1. ISPRS Vaihingen Dataset

Ablation studies were conducted on the Vaihingen dataset to evaluate the effectiveness of SDA-Net, focusing on three key components: the Dual-Stream Encoder, Spatial Information Optimization Module (SIOM), and the Adaptive Global Attention Fusion Module (AGAFM).

**Figure 11:**
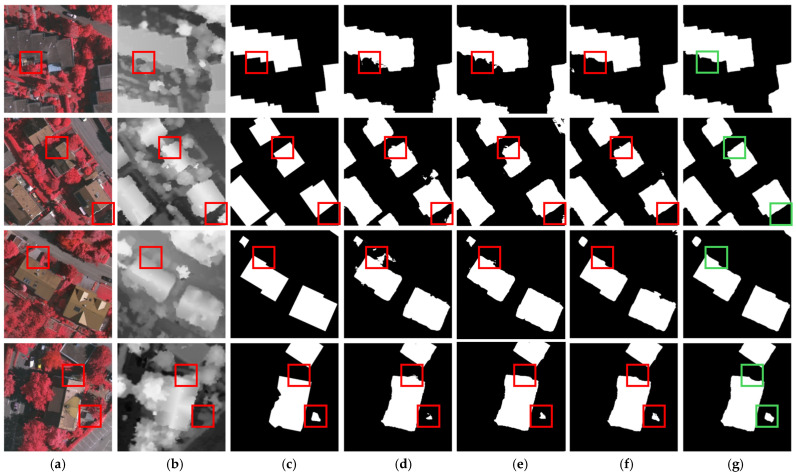
Visualization results of ablation study on the ISPRS Vaihingen Dataset. (**a**) IRRG images. (**b**) DSM images. (**c**) Labels. (**d**) Axial self-attention. (**e**) Dual-Stream. (**f**) Dual-Stream + SIOM. (**g**) SDA-Net.

The experimental framework employed U-shaped architectures incorporating three distinct encoder configurations: ResNet50, axial self-attention (Axial SA), Transformer (ViT-12), and the proposed Dual-Stream encoder. Following this baseline evaluation, the SIOM was implemented to investigate its efficacy in multi-modal feature alignment by suppressing feature redundancy and noise interference. Subsequently, the AGAFM was integrated to dynamically bridge the semantic gap between multi-modal multi-scale features. Comprehensive experimental validation, as documented in Table 4 and Figure 11, reveals progressive performance enhancements through sequential integration of these components within the SDA-Net architecture. The quantitative results and visualizations demonstrate statistically significant improvements in key metrics at each developmental stage of the network’s construction.

The experimental results, as presented in Table 4, include not only the statistics of accuracy, recall, F1-score, and IoU but also a comprehensive evaluation of the computational complexity and practical efficiency of different models through three key metrics: FLOPs (F), Parameters (P), and Inference Time (T). FLOPs, measured in GFLOPs (G), are utilized to quantify the theoretical computational complexity required for a single forward pass of the model. Parameters, reported in millions (M), reflect the total number of learnable weights, directly determining the model’s memory footprint and storage demands. Inference Time, defined as the duration in seconds (s) for processing a single 512 × 512-pixel image, is calculated by averaging the latency across batches.

According to the ablation study results presented in Table 4, substituting ResNet50 with an axial self-attention branch as the feature extraction encoder yields significant performance improvements, achieving absolute gains of 2.64% in F1 score and 4.54% in IoU. The notable 3.42% recall enhancement confirms the axial self-attention’s superior capability in capturing comprehensive global contextual features, which substantially improves building pixel identification accuracy. The Transformer encoder significantly increases FLOPs and parameters while extending the inference time by 0.61 s, and achieves only marginal performance gains of 0.3% in F1-score and 0.06% in accuracy compared to the axial self-attention mechanism. In contrast, the axial self-attention decomposition effectively mitigates computational complexity while preserving comparable segmentation performance, demonstrating its superior capability to balance computational efficiency with feature representation capacity through axis-wise attention factorization. The Dual-Stream encoder architecture, which synergistically integrates ResNet50’s local feature extraction with axial self-attention’s global modeling, delivers additional performance increments of 0.75% F1 score and 1.31% IoU compared to the standalone axial self-attention implementation. This hybrid approach achieves Precision enhancement while maintaining comparable recall levels, indicating ResNet50’s complementary role in preserving critical local structural details. This architecture, due to its involvement in the alignment and fusion of features from different branches, significantly increases FLOPs while maintaining parameters and inference time at a lightweight level. The implementation of SIOM at the network’s initial stage establishes spatial alignment for multi-modal features through coordinate optimization, which in turn facilitates joint representation refinement and systematic discovery of cross-modal complementarity. Notably, the introduction of SIOM incurs almost no additional parameters and only a slight increase in inference time, yet it achieves significant performance improvements. Experimental results demonstrate 2.53% recall enhancement with concurrent improvements of 1.12% F1 score and 2.02% IoU metrics. The marginal 0.4% Precision reduction likely stems from the module’s noise suppression mechanism potentially attenuating sensitivity to subtle feature variations. Despite the computational overhead introduced by graph convolution operations, the incorporation of AGAFM significantly enhances model performance through dynamic semantic gap modulation, yielding notable improvements of 1.46% in F1-score and 2.69% in IoU. These improvements substantiate the framework’s capability to achieve adaptive fusion of local and global features via context-aware feature recalibration.

The visualization results of the ablation experiments are presented in Figure 11. As shown in Figure 11d,e, the adoption of the Dual-Stream encoder enables more accurate extraction of building contours compared to the standalone axial self-attention encoder. After introducing SIOM, effective suppression of noise in multi-modal data are achieved, as demonstrated in the first and second rows (e) and (f). Finally, the progressive application of the AGAFM dynamically bridges semantic gaps and optimizes the balance between global and local information, generating high-fidelity fused features with enhanced discriminative power for precise extraction of detailed building features, as shown in the third and fourth rows (f) and (g).

In summary, the progressive integration of axial self-attention, Dual-Stream encoder, SIOM, and AGAFM has enhanced model performance across metrics while demonstrating their efficacy in building extraction tasks, culminating in a framework that achieves a balanced trade-off between extraction accuracy and computational efficiency.

#### 4.4.2. DFC23 Track2 Dataset

**Figure 12:**
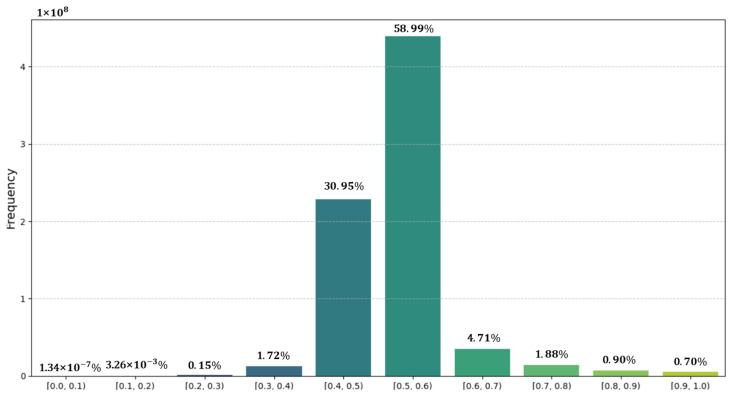
Weight value distribution intervals.

To better illustrate the selection of the threshold T in the SIOM, we statistically analyzed the numerical distribution of pre-classification weights, as shown in the histogram in Figure 12. The results reveal that the majority of weight values (89.94%) are concentrated within the interval [0.4, 0.6], while values below 0.2 or above 0.8 are extremely sparse. This implies that setting T in these peripheral regions would result in nearly all data being categorized into a single class, effectively disabling the SIOM. Consequently, experiments were conducted across T ∈ [0.3, 0.8] to systematically evaluate the impact of T on model performance, with results summarized in Table 5.

The empirical analysis demonstrates a clear performance trend as follows: when T ∈ [0.45, 0.55], the model achieves peak F1-score of 91.35% and IoU of 84.08%, indicating robust discrimination of positive samples within this range. However, exceeding T = 0.55 leads to a marked a 0.44% drop in Precision, suggesting that overly lenient thresholds over-suppress critical features, albeit maintaining high recall by tolerating redundant information. Conversely, thresholds below T = 0.45 significantly constrain recall, with a reduction of 0.90%, as excessively strict filtering retains excessive noise. Based on the comprehensive experimental results, T ∈ [0.45, 0.55] is identified as the optimal range. If further refinement of the optimal value is required, more detailed numerical distribution statistics or the introduction of an adaptive mechanism to dynamically adjust T could be employed. For the sake of balancing efficiency, we directly selected T = 0.5 as the threshold for weight decomposition in our experiments.

In order to further validate the contribution of the Adaptive Global Attention Fusion Module (AGAFM) to model performance, we conducted a comprehensive ablation study analyzing multi-scale feature fusion strategies on the DFC23 Track2 dataset.

To systematically evaluate fusion mechanisms, we implemented a unified Dual-Stream encoder architecture equipped with the SIOM. Comparative analysis encompassed six fusion strategies: element-wise summation (Sum), concatenation (Cat), isolated Channel Attention (CA), isolated Spatial Attention (SA), a cascaded Spatial-Channel Attention without integration of the adaptive global aggregation module (CSA) and the proposed AGAFM.

Experimental results are summarized in Table 6 and demonstrate the superior performance of AGAFM across all evaluation metrics. The proposed AGAFM achieves significant improvements over existing fusion approaches, attaining 5.55% and 8.94% absolute gains in F1 score and IoU, respectively, compared to element-wise summation. Relative to concatenation operations, AGAFM exhibits 3.42% and 5.63% enhancements in these core metrics. When benchmarked against channel attention (CA) and spatial attention (SA) mechanisms, the module maintains consistent performance advantages with 2.57%/4.25% and 3.12%/5.17% improvements in F1/IoU metrics. Notably, AGAFM also surpasses the cascaded spatial-channel attention architecture (CSA) by 1.93% F1 score and 3.22% IoU, confirming its effectiveness in multi-scale multi-modal feature fusion through global context aggregation for attention weight optimization. These comprehensive comparisons conclusively validate the technical contributions of AGAFM’s adaptive global aggregation mechanism.

**Figure 13:**
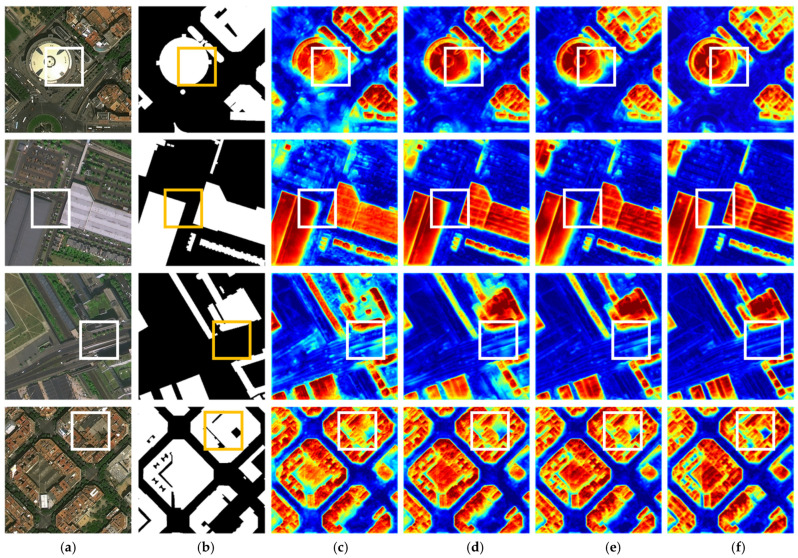
Visualization results of fusion methods. (**a**) RGB images. (**b**) Labels. (**c**) Sum. (**d**) CA. (**e**) CSA. (**f**) AGAFM.

To better visualize the feature fusion effect, intermediate features were extracted during testing and presented as heatmaps in Figure 13. The first and second rows demonstrate that it is evident that the feature fusion guided by AGAFM restores the most accurate contour structures. In the third row, it is notable that the high-frequency features with interference have been effectively suppressed. The fourth row showcases regions with complex backgrounds, where the AGAFM fusion successfully focuses on the fine-grained building features. AGAFM balances semantic discrepancies during multi-scale cross-modal feature fusion through flexible attention, selectively leveraging different features to complement each other. This approach not only prevents excessive interference from high-frequency information on global features but also avoids the overshadowing of key structural contour features by global information. This capability enhances the model’s performance in building extraction tasks, enabling more precise and reliable results even in challenging environments.

## 5. Discussion and Conclusions

This paper proposes SDA-Net, a novel building extraction network designed for multi-modal remote sensing data, which addresses critical challenges in heterogeneous feature alignment and semantic gaps across multi-scale, multi-modal inputs. SDA-Net outperforms existing models through three key structural designs: The Spatial Information Optimization Module (SIOM) enables precise cross-modal spatial alignment and noise suppression by decomposing features into structural and semantic components, effectively mitigating domain discrepancies in multi-modal data. The Dual-Stream encoder, integrating axial self-attention and CNNs, captures both global contextual dependencies and fine-grained local details, significantly enhancing robustness in complex urban environments with dense, overlapping structures. The Adaptive Global Attention Fusion Module (AGAFM) dynamically resolves semantic conflicts during multi-scale feature fusion, enabling adaptive weighting of cross-modal contributions. Extensive experiments on the ISPRS Potsdam, Vaihingen, and DFC23 Track2 datasets demonstrate SDA-Net’s superior performance, achieving state-of-the-art results in Precision, Recall, F1 score, and IoU. Ablation studies validate the effectiveness of each component, particularly highlighting SIOM’s role in multi-modal feature alignment and AGAFM’s capability in adaptive feature fusion.

While SDA-Net has made significant progress in multi-modal feature fusion, current building extraction methods still face fundamental limitations, including the inherent lack of capability to infer critical three-dimensional urban attributes, such as building height and floor count, which are essential for comprehensive urban digital twin modeling. Although UAV-derived photogrammetric data can partially address this gap by providing high-resolution 3D point clouds through vertical structure reconstruction, such single-source solutions remain constrained by limited spatial coverage and spectral constraints. This underscores the necessity of synergistic fusion between UAV and satellite remote sensing data—a highly promising direction that can integrate centimeter-level geometric precision from UAVs with spectral–temporal information from satellites, ultimately enabling holistic urban characterization across multiple dimensions. Moreover, the detection of vegetation-occluded buildings remains a critical challenge for automated systems, where human interpreters still outperform algorithms through contextual reasoning. To bridge this gap, a promising direction worth exploring is the integration of generative models with hierarchical attention mechanisms to enhance reasoning capabilities.

## Figures and Tables

**Table 1:** Comparative Experiments of SDA-Net on the ISPRS Potsdam dataset.

Method	Precision (%)	Recall (%)	F1 (%)	IoU (%)
SERNet	96.06	94.56	95.30	91.03
RDFNet	93.10	93.91	93.50	87.78
RedNet	96.81	90.23	93.40	87.62
MMFNet	96.03	96.64	96.33	92.93
ADEUNet	97.60	96.77	97.18	94.52
SA-Gate	97.42	96.46	96.94	94.06
CMGFNet	98.03	96.93	97.48	95.08
TransUnet	95.84	95.53	95.68	91.37
ST-Unet	96.79	96.21	96.50	93.24
SDA-Net	**98.10**	**97.22**	**97.66**	**95.42**

Bold values indicate the best performance.

**Table 2:** Comparative experiments of SDA-Net on the ISPRS Vaihingen dataset.

Method	Precision (%)	Recall (%)	F1 (%)	IoU (%)
SERNet	90.63	92.68	91.65	84.58
RDFNet	96.60	93.88	95.22	90.88
RedNet	95.99	95.32	95.65	91.66
MMFNet	95.50	93.29	94.39	89.36
ADEUNet	**97.25**	94.92	96.07	92.44
SA-Gate	96.83	94.64	95.72	91.80
CMGFNet	96.98	94.97	95.96	92.24
TransUnet	95.58	93.77	94.67	89.87
ST-Unet	96.41	95.52	95.97	92.25
SDA-Net	97.08	**96.05**	**96.56**	**93.35**

Bold values indicate the best performance.

**Table 3:** Comparative experiments of SDA-Net on the DFC23 Track2 dataset.

Method	Precision (%)	Recall (%)	F1 (%)	IoU (%)
SERNet	87.21	84.08	85.62	74.85
RDFNet	89.58	83.51	86.44	76.10
RedNet	87.20	87.72	87.46	77.71
MMFNet	91.37	89.72	90.54	82.71
ADEUNet	87.62	86.36	86.98	76.97
SA-Gate	88.79	87.39	88.08	78.71
CMGFNet	91.23	79.06	84.71	73.48
TransUnet	87.16	85.53	86.34	75.97
ST-Unet	90.38	86.19	88.24	78.95
SDA-Net	**91.69**	**91.02**	**91.35**	**84.08**

Bold values indicate the best performance.

**Table 4:** Ablation study of SDA-Net on the ISPRS Vaihingen dataset.

Encoder	SIOM	AGAFM	Precision (%)	Recall (%)	F1 (%)	IoU (%)	F (G)	P (M)	T (s)
ResNet50	-	-	92.10	89.12	90.59	82.79	**44.91**	48.41	**0.094**
Axial SA	-	-	93.93	92.54	93.23	87.33	52.68	**18.00**	0.151
Transformer	-	-	93.99	93.07	93.53	87.84	114.52	89.06	0.761
Dual-Stream	-	-	95.68	92.34	93.98	88.64	189.06	53.91	0.279
Dual-Stream	√	-	95.34	94.87	95.10	90.66	189.13	53.92	0.422
Dual-Stream	√	√	**97.08**	**96.05**	**96.56**	**93.35**	189.14	62.27	1.331

Bold values indicate the best performance.

**Table 5:** Ablation study of SIOM on the DFC23 Track2 dataset.

T	Precision (%)	Recall (%)	F1 (%)	IoU (%)
0.3	91.61	89.40	90.49	82.63
0.4	91.37	89.94	90.65	82.90
0.45	**91.85**	90.84	91.34	84.06
0.5	91.69	91.02	**91.35**	**84.08**
0.55	91.34	**91.38**	91.35	84.08
0.6	90.90	91.26	91.08	83.63
0.7	90.77	91.00	90.88	83.30
0.8	90.65	90.80	90.72	83.03

Bold values indicate the best performance.

**Table 6:** Ablation study of AGAFM on the DFC23 Track2 dataset.

Fusion Method	Precision (%)	Recall (%)	F1 (%)	IoU (%)
Sum	86.50	85.12	85.80	75.14
Cat	88.43	87.43	87.93	78.45
CA	89.31	88.26	88.78	79.83
SA	88.42	88.01	88.22	78.91
CSA	89.62	89.21	89.42	80.86
AGAFM	**91.69**	**91.02**	**91.35**	**84.08**

Bold values indicate the best performance.

## Data Availability

The datasets utilized in this study are publicly available, with details as follows: ISPRS Potsdam dataset is available from the International Society for Photogrammetry and Remote Sensing (ISPRS) benchmark platform at https://www.isprs.org/education/benchmarks/UrbanSemLab/2d-sem-label-potsdam.aspx; ISPRS Vaihingen dataset is available from the ISPRS benchmark platform at https://www.isprs.org/education/benchmarks/UrbanSemLab/2d-sem-label-vaihingen.aspx; Descriptions and access methods of DFC23 track2 dataset are provided in the cited publication with permanent DOI: https://doi.org/10.1109/MGRS.2023.3240233.
